# PAINT using proteins: A new brush for super‐resolution artists

**DOI:** 10.1002/pro.3953

**Published:** 2020-09-25

**Authors:** Curran Oi, Simon G. J. Mochrie, Mathew H. Horrocks, Lynne Regan

**Affiliations:** ^1^ Department of Molecular Biophysics and Biochemistry Yale University New Haven Connecticut USA; ^2^ Integrated Graduate Program in Physical and Engineering Biology Yale University New Haven Connecticut USA; ^3^ Department of Physics Yale University New Haven Connecticut USA; ^4^ School of Chemistry The University of Edinburgh Edinburgh UK; ^5^ Institute of Quantitative Biology, Biochemistry and Biotechnology, Centre for Synthetic and Systems Biology, School of Biological Sciences University of Edinburgh Edinburgh UK

**Keywords:** coiled coil, fluorescence microscopy, live cell imaging, peptide, protein, super‐resolution, TPR

## Abstract

PAINT (points accumulation for imaging in nanoscale topography) refers to methods that achieve the sparse temporal labeling required for super‐resolution imaging by using transient interactions between a biomolecule of interest and a fluorophore. There have been a variety of different implementations of this method since it was first described in 2006. Recent papers illustrate how transient peptide–protein interactions, rather than small molecule binding or DNA oligonucleotide duplex formation, can be employed to perform PAINT‐based single molecule localization microscopy (SMLM). We discuss the different approaches to PAINT using peptide and protein interactions, and their applications in vitro and in vivo. We highlight the important parameters to consider when selecting suitable peptide–protein interaction pairs for such studies. We also note the opportunities for protein scientists to apply their expertise in guiding the choice of peptide and protein pairs that are used. Finally, we discuss the potential for expanding super‐resolution imaging methods based on transient peptide–protein interactions, including the development of simultaneous multicolor imaging of multiple proteins and the study of very high and very low abundance proteins in live cells.

## 1 INTRODUCTION

**FIGURE 1:**
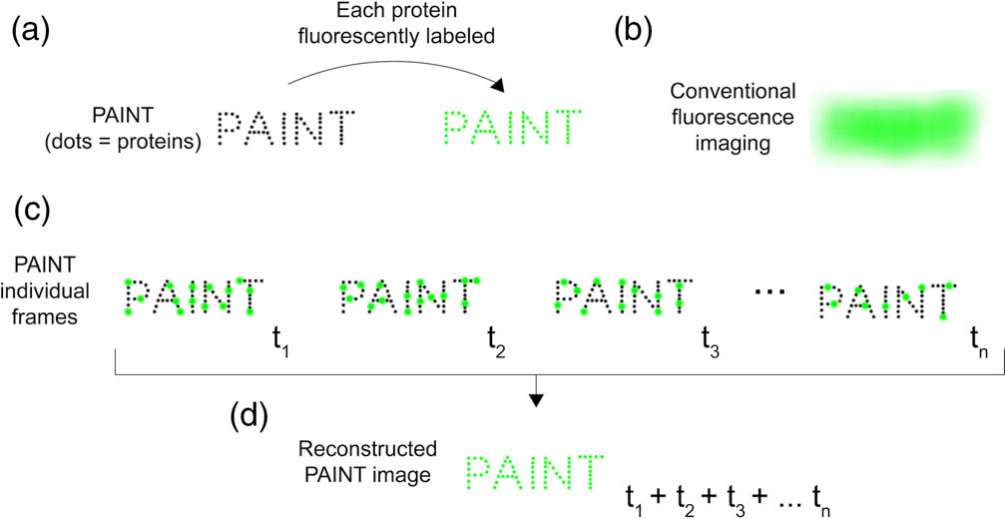
Cartoon illustration of the principle of the PAINT method of SMLM. PAINT achieves super‐resolution by summing sparse, temporally‐separated localization events. (a) A biomolecular structure “PAINT” (dimensions of the order of 500 × 2000 nm) composed of multiple proteins. Individual proteins are shown as black dots. If each protein is directly fused to a fluorescent molecule (green dots), (b) conventional fluorescence imaging cannot resolve individual fluorophores, so the PAINT structure is fluorescent, but individual proteins cannot be visualized—because the proteins are too close together to be resolved by diffraction‐limited microscopy. (c) SMLM by PAINT. The proteins in the biomolecular structure are not directly fused to a fluorescent molecule. They are only visible when a fluorescent molecule transiently binds to any of them, resulting in intense bursts of fluorescence (green spots). Such data are collected iteratively over time (*t*_1_, *t*_2_, *t*_3_, … *t*_*n*_). At each timepoint, a different subset of the proteins is bound to the fluorescent molecule. (d) The localization events collected at each timepoint (*t*_1_, *t*_2_, *t*_3_, … *t*_*n*_) in panel (c) are summed to generate a final super‐resolution image, in which the location of each protein can now be resolved

Recent papers describe the successful use of transient peptide–protein interactions to perform super‐resolution microscopy,^1^, ^2^, ^3^ in particular as a new way to implement the method known as points accumulation for imaging in nanoscale topography (PAINT)^4^ (Figure 1).

These studies demonstrate that different implementations of PAINT, employing protein–peptide interaction pairs, enable high resolution single‐molecule localization microscopy (SMLM) in vitro, in fixed cells, and inside live cells. These studies also highlight an exciting opportunity for protein scientists to develop new tools for super‐resolution imaging, by identifying natural peptide–protein pairs with desirable characteristics, or by creating new ones.

**FIGURE 2:**
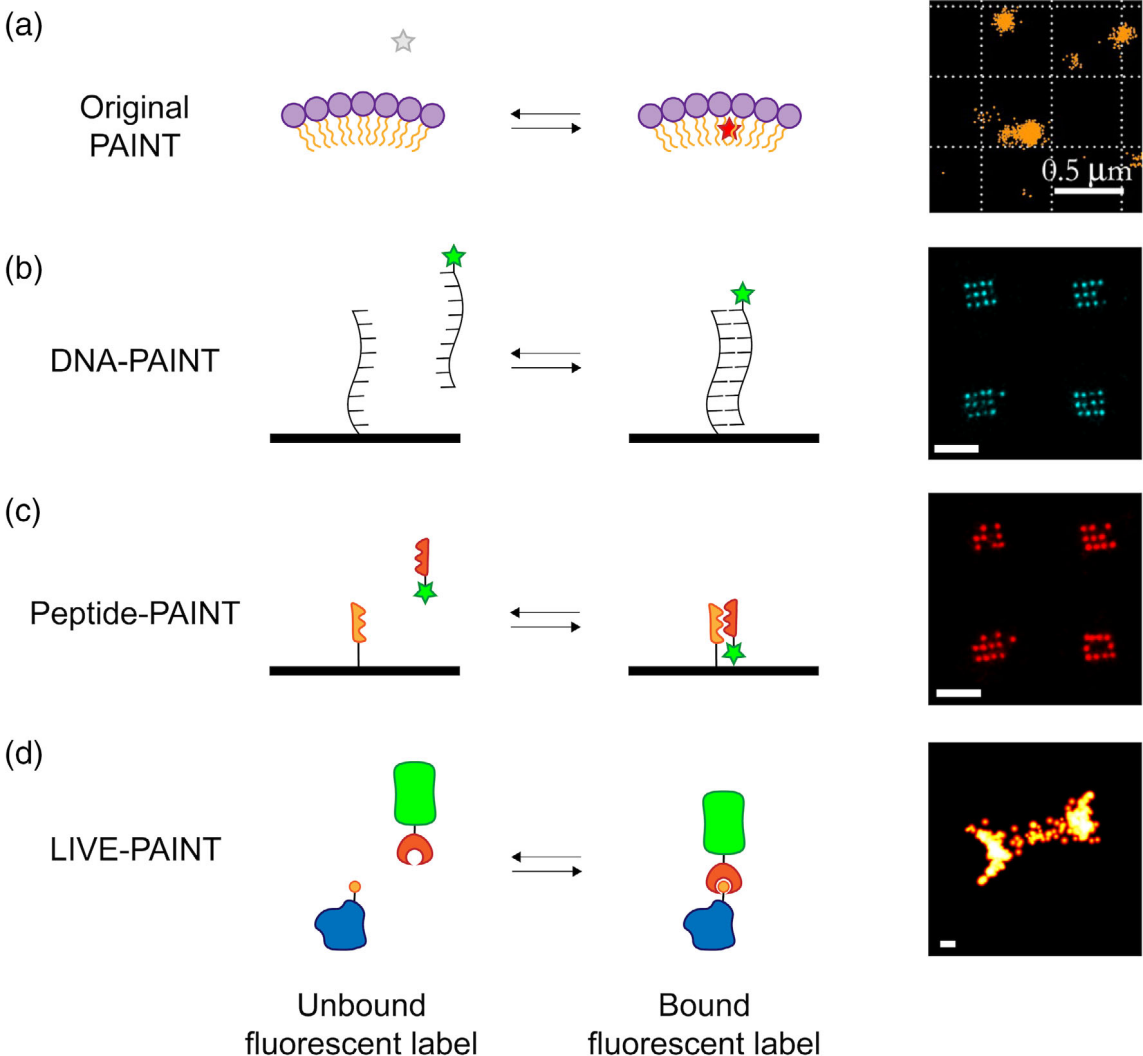
Cartoon illustration of different PAINT methods. (a) “Original PAINT”. Left: a cartoon representation of a small molecule dye which is nonfluorescent in aqueous solution (grey star) but which fluoresces (red star) when it transiently interacts with the hydrophobic lipid membrane of the LUV (purple circles represent polar headgroups, yellow tails represent the aliphatic tails). Right: image of LUVs imaged using Nile red modified from Sharonov et al.^4^ [copyright (2006) National Academy of Sciences]. (b) DNA‐PAINT. Left: cartoon representation in which the short ssDNA oligonucleotide to be imaged (for example part of a DNA origami surface array) is shown as a black strand. The complementary ssDNA oligonucleotide is shown as a black strand attached to a fluorescent dye (green star). Right: DNA origami nanostructures imaged using DNA‐PAINT, reproduced from Eklund et al.^1^ (copyright Creative Commons Attribution 4.0 International license: https://creativecommons.org/licenses/by/4.0/). (c) Peptide‐PAINT. Left: cartoon representation in which the protein to be imaged is fused to a peptide (orange saw‐ tooth). The protein is visualized by the interaction of that peptide with a protein that binds it (red sawtooth) fused to a fluorescent dye (green star). Right: DNA origami nanostructures imaged using Peptide‐PAINT, reproduced from Eklund et al.^1^ (copyright Creative Commons Attribution 4.0 International license: https://creativecommons.org/licenses/by/4.0/). (d) LIVE‐PAINT. Left: cartoon illustration in which the protein to be imaged (blue) is fused, at the gene level, to a peptide (orange circle). The protein is visualized by the interaction of that peptide with a protein that binds to it (red crescent), fused to a fluorescent protein (green barrel). Any fluorescent protein can be used. Oi et al. used the bright mNeonGreen. Right: image of the septum in live *Saccharomyces cerevisiae*, obtained by labeling Cdc12p and imaged using LIVE‐PAINT. Scalebars are 500 nm (a) and 100 nm (b–d). All images shown in panels (a–d) were acquired using TIRF

PAINT‐based strategies rely on transient interactions between a fluorescent molecule and a target biomolecule (Figure 2a). Sharonov and Hochstrasser demonstrated the first implementation of PAINT by imaging large unilamellar vesicles (LUVs). They used the dye Nile red,^4^ a small environmentally‐sensitive fluorophore which exhibits intense red fluorescence in a hydrophobic environment, but minimal fluorescence in an aqueous environment. When a molecule of Nile red transiently interacts with the LUV membrane, localized bursts of high intensity fluorescence are observed. Because these fluorescent bursts are spatially and temporally separated, the center of each can be identified, and a super‐resolution image of the membrane constructed by summing these individual localizations (Figure 1a–d). PAINT using different small molecule fluorophores has also been used to map the hydrophobic surfaces of different amyloid aggregates,^5^, ^6^, ^7^, ^8^ again taking advantage of both the transient interaction and increase in fluorescence upon binding in a hydrophobic environment.

A key advantage of PAINT‐based methods, compared to other SMLM approaches, is their ability to circumvent the issue of fluorescent probes photobleaching over time, which is an inevitable consequence of irradiation by the excitation laser. In approaches employing covalently‐bound fluorescent probes, the number of emitting fluorophores decreases as the experiment proceeds, thus progressively fewer localization events are recorded as time continues. Eventually all the fluorophores are bleached. The duration of data acquisition and the number of localizations is thus strictly limited. By contrast, in PAINT‐based approaches, because the interaction between the biomolecule and the fluorescent probe is transient, bound but bleached fluorescent molecules will be continually replaced by exchanging with unbleached, unbound molecules. Data acquisition can thus continue beyond the time scale for bleaching, enabling extended data accumulation, consequently generating higher resolution images. This also allows dim bursts that are localized with a low accuracy to be removed during analysis, since there are many more localizations of higher precision. An additional advantage of PAINT methods is that they do not require a photoconvertible fluorophore. There are thus many more small molecule fluorophores or fluorescent proteins, with a greater range of emission wavelengths, to choose from.

## 2 ENCODING SPECIFICITY USING DNA‐PAINT

A limitation of PAINT using small molecules is the lack of specificity in their interaction. The development of DNA‐PAINT provided a strategy for PAINT‐type SMLM visualization, but via a highly specific interaction. DNA‐PAINT is an elegant technique which uses two short complementary oligonucleotides, one attached to a biomolecule of interest and one labeled with a fluorescent dye^9^ (Figure 2b). These oligonucleotides interact transiently, resulting in bursts of fluorescence, just as in the original small‐molecule PAINT experiments. In this implementation of the method, however, the transient interaction is highly specific, dictated by the sequence of the two complementary DNA strands. DNA‐PAINT has been widely used to image DNA origami type structures in vitro.^9^, ^10^, ^11^, ^12^, ^13^, ^14^, ^15^, ^16^, ^17^, ^18^, ^19^, ^20^

The enormous advantage of DNA‐PAINT is that it is relatively straightforward to manipulate the specificity and affinity of the two interacting ssDNA strands. In more elaborate implementations, involving a ssDNA attached to a nanobody or aptamer for example, DNA‐PAINT has been used to image proteins within fixed, permeabilized cells.^10^, ^15^, ^16^, ^17^, ^18^, ^21^, ^22^ These examples, however, make clear one of the main limitations of DNA‐PAINT: It cannot be used inside live cells.

In its original implementation, DNA‐PAINT was constrained by the intrinsically slow binding rate of complementary ssDNA oligonucleotides, leading to long image acquisition times at the solution concentrations needed to avoid significant background from unbound labeled oligonucleotide. It could take hours to obtain high resolution images.^19^ Recently, however, Strauss and Jungmann have shown that on‐rates can be increased about a hundred‐fold by using multiple concatenated repeats of a short DNA sequence.^19^ This modification of the method enables sufficient data for a 20 nm resolution image to be acquired in minutes.

## 3 PROTEIN‐BASED PAINT METHODS

Protein–protein on rates can be enhanced by favorable electrostatic interactions, and can therefore be much faster than the association of two negatively charged, albeit complementary in sequence, oligonucleotides.^23^, ^24^, ^25^

Protein‐based PAINT methods (Figure 2c,d) therefore have the potential to provide a straightforward route to increased on‐rates. Indeed, in a method they named Peptide‐PAINT, Eklund et al. showed that using peptide–peptide interactions can increase imaging speeds of a DNA origami array two‐fold, relative to imaging the same array using ssDNA‐ssDNA interactions, in DNA‐PAINT.^1^

Eklund et al. started with the E_3_/K_3_ coiled coil pair of 21 amino acid (aa) peptides, where each E unit is a negatively charged 7 aa “heptad repeat” and each K unit is a positively charged 7 aa “heptad repeat” peptide.^26^, ^27^, ^28^ Keeping the length of the negatively charged peptide constant, Eklund et al. explored the effect of decreasing the length of the K peptide on coiled coil stability. They chose to work with K peptides of 18 or 19 aa, which interact with the E peptide with dissociation constants of 1.7 μM and 81 nM, respectively. These dissociation constants are similar to the dissociation constants of DNA duplexes that have previously been effective in DNA‐PAINT.

The majority of the testing of Peptide‐PAINT was in vitro, in the context of a DNA origami array, which allowed a direct comparison between the behavior of the peptide pair with that of a DNA duplex. It proved possible to image the DNA origami surface using Peptide‐PAINT in a similar fashion to using DNA‐PAINT. An advantage of Peptide‐PAINT is faster “on‐rates” than for conventional DNA‐PAINT. With Peptide‐PAINT, the mean dark time between fluorescent bursts for a given binding site is approximately 30 s, compared with approximately 70 s for DNA‐PAINT.

## 4 IMAGING PROTEINS IN CELLS USING PROTEIN‐BASED PAINT

**FIGURE 3:**
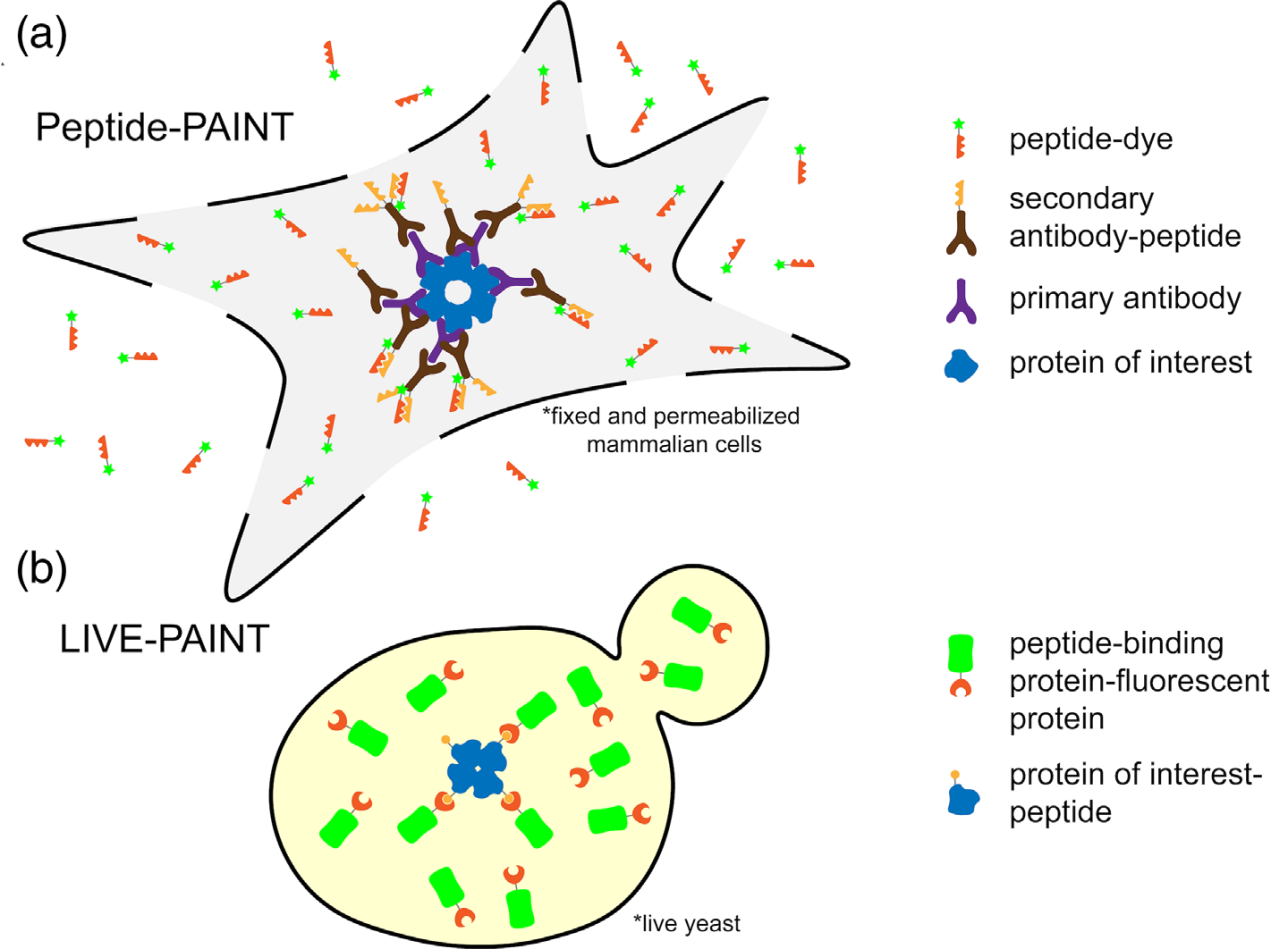
Cartoon representations of using Peptide‐PAINT in fixed, permeabilized mammalian cells (a) and LIVE‐PAINT inside live yeast (b). (a) The protein of interest (blue) is bound by a primary antibody (purple). A secondary antibody (brown), which binds to the primary antibody, is attached to one or more peptides (orange sawtooth). A peptide (red sawtooth) that interacts with the antibody‐linked peptide is synthesized with a fluorescent dye (green star) attached. Cells are fixed and permeabilized and the peptide‐dye fusion (red sawtooth‐green star) is added exogenously and can diffuse in and out of the cell. Excess antibodies and fluorescently labeled peptide can be washed out prior to imaging in TIRF, which further decrease the background. (b) The protein of interest (blue) is fused to a peptide (orange circle), at the gene level, and integrated into the chromosome. A peptide‐binding protein, comprising the recognition element for the peptide (red crescent) is fused to a fluorescent protein (green barrel), at the gene level, and integrated into the chromosome. Labeling is performed inside live cells, with the expression level of the labeling protein controlled. Background from unbound labeling protein is reduced by data acquisition in TIRF

Eklund et al. showed that the Peptide‐PAINT method has the potential to be used in fixed permeabilized cells. In addition to requiring that a cell is fixed and permeabilized, in its current implementation, Peptide‐PAINT also requires an antibody or (antibody equivalent) against any protein of interest and chemical coupling of a peptide to a secondary antibody (Figure 3a). The stoichiometry between the antibody and the coiled coil strand is variable, because the conjugation attaches the coiled coil peptide to any accessible primary amine on the antibody.^1^ The complementary strand of the coiled coil duplex is conjugated to a fluorophore and added exogenously to the fixed and permeabilized cells. This strategy has the disadvantage of increasing the distance between the protein of interest and the fluorophore, thus decreasing the precision of localization of the protein of interest. It has been previously shown that conjugating a fluorophore to a primary antibody increases the distance between the target and fluorophore by ~12.5 nm.^29^ Using both primary and secondary antibodies will increase this distance between target molecule and fluorophore even more, likely to more than 20 nm.

Other key developments in using peptide–peptide or peptide–protein interactions for PAINT‐type super‐resolution imaging have focused on the important advantage that they can be genetically encoded and thus work inside live cells.

The idea of fluorescently labeling a protein of interest via a non‐covalent interaction with a fluorescent molecule, has previously been described for traditional fluorescence imaging. For example, Pratt et al. fused a 5 aa peptide to the protein of interest via an 8 aa linker sequence, which was then visualized in live *E*. *coli*, by its interaction with a 120 aa tetratricopeptide repeat (TPR) domain fused to a fluorescent protein.^30^, ^31^ Related work by Hinrichsen et al. showed that a similar method could be used to fluorescently label a membrane protein post‐translationally in live yeast,^32^ thus avoiding the perturbation of function associated with direct fusion of a fluorescent protein to a membrane protein.

Perfilov et al. showed that different versions of the E_3_/K_3_ peptides (containing point mutations) could be used to perform super‐resolution imaging in live cells.^3^ In this example, they used a peptide attached to a photo‐convertible fluorescent protein, and used photoactivated localization microscopy (PALM) to obtain data for a super‐resolution image. Although a peptide–protein interaction is used in this work, it differs from the work of Eklund et al.^1^ and Oi et al.,^2^ in not employing a PAINT approach to data acquisition. The work is analogous to the peptide‐protein pair mediated fluorescence labeling of Pratt et al. but with PALM super‐resolution imaging rather than diffraction limited imaging.

**FIGURE 4:**
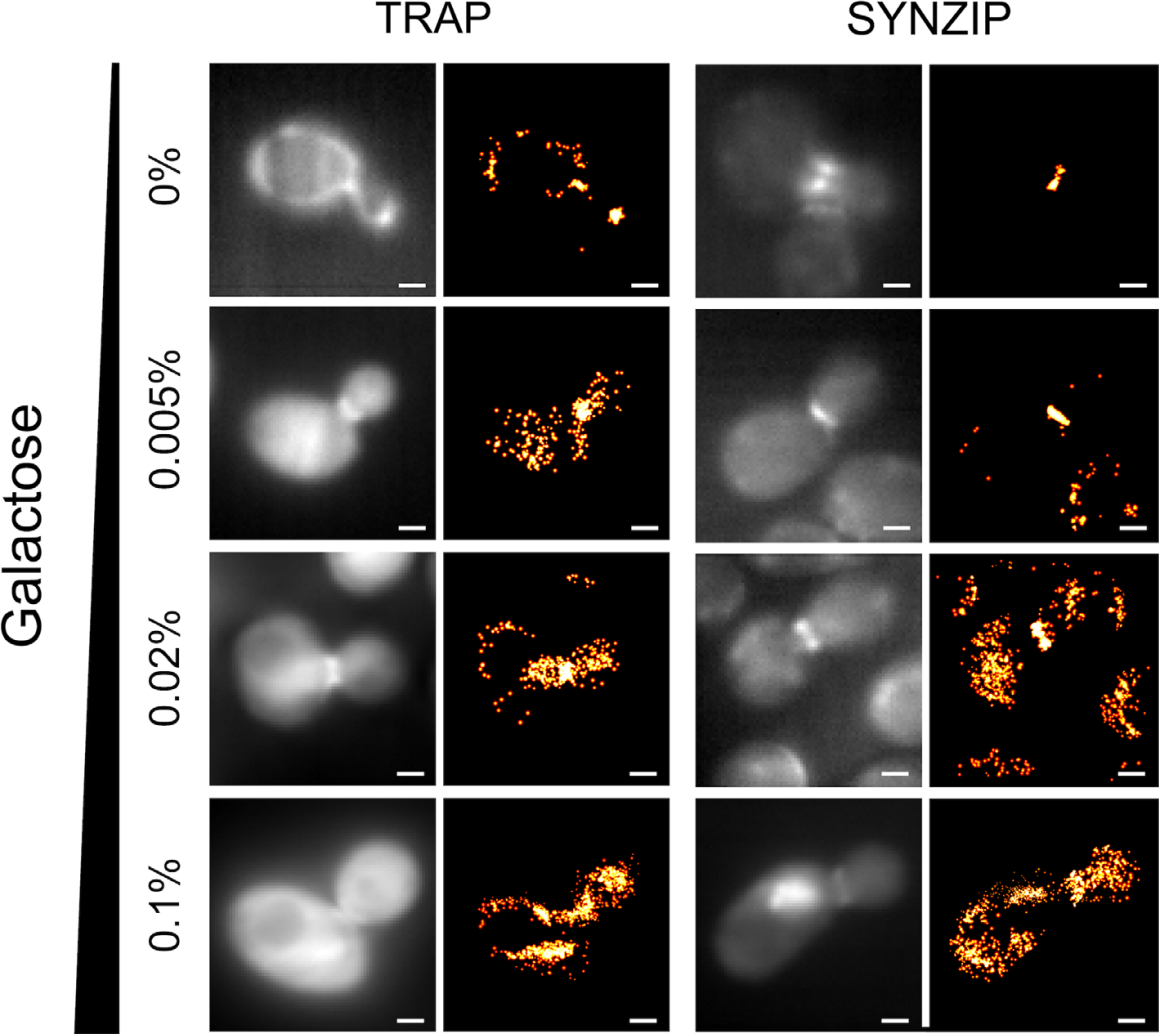
Varying either the fluorescent protein expression level or the peptide–protein interaction pairs changes the number of localization events at the yeast bud neck during cell division. Pairs of diffraction‐limited and super‐resolution images are shown for Cdc12p‐MEEVF + TRAP4‐mNG (left) and Cdc12p‐SYNZIP18 + SYNZIP17‐mNG (right), at different concentrations of galactose (as indicated on the left). Expression level of the fluorescent construct was shown to be directly proportional to galactose concentration. Scale bars are 1 μm. Figure reproduced from Oi et al.^2^ (copyright Creative Commons Attribution 4.0 International license: https://creativecommons.org/licenses/by/4.0/)

Oi et al. investigated the use of peptide–protein interactions to perform super‐resolution imaging inside live yeast cells, naming this method LIVE‐PAINT^2^ (Figures 2d and 3b). All the imaging was performed on live cells, in which the chromosome was engineered to express the desired proteins. In this work, the protein of interest was fused to a peptide (either a 5 aa peptide for the TPR interaction, or a 42 aa peptide for the coiled coil interaction) via an 8 aa linker sequence. They used a hetero‐dimeric antiparallel coiled coil,^33^ or a peptide‐TPR pair,^34^ having observed that that fusion of highly charged peptides^35^ to their test protein of interest (Cdc12p) resulted in aberrant cell morphology and growth. They explored how the labeling efficacy changes with the dissociation constant of the peptide–protein pair and the amount of the labeling protein expressed (Figure 4). The key difference between LIVE‐PAINT and DNA‐PAINT and Peptide‐PAINT is that in LIVE‐PAINT all the components are genetically encoded and expressed within the cell.

## 5 IMPORTANT REQUIREMENTS FOR A GOOD PROTEIN–PEPTIDE OR PEPTIDE–PEPTIDE PAIR FOR IMAGING USING PAINT METHODS

When considering which peptide–peptide or peptide–protein interactions are suitable for PAINT imaging, there are several important considerations. The peptide–protein pair must be specific. The 1:1 heterodimer should be overwhelmingly favored over all other states, such as higher order oligomers. There should be minimal or no homodimer formation by either component, and neither component should interact significantly with any cellular protein. The peptide fused to the protein of interest should be small, to avoid perturbing that protein's function. The peptide–protein interaction should be of a suitable strength to function as desired. It should not be too tight, because the PAINT approach relies on transient interactions and exchange of the bound state with the unbound pool. Operationally, a dissociation constant of about 1 μM is desirable,^1^, ^3^, ^9^ although the on‐rate and off‐rate of binding is more important than the *K*_*d*_.

It is desirable for the peptide–protein interaction to have a relatively fast off‐rate. Most DNA‐PAINT experiments use interactions with an off‐rate of approximately 1 s^−1^.^9^ In the original DNA‐PAINT experiments, the authors note that the off‐rate of the DNA strands is not highly dependent on the length of the DNA strands used, suggesting that there is little scope for modulating the off‐rate for interactions between two short DNA strands.^9^ In principle, the desirable off‐rate for PAINT experiments is one which allows sufficient photons to be observed during a localization event to achieve “good” resolution. In DNA‐PAINT experiments, exposure times of 100 ms are typically used^9^, ^19^ and in LIVE‐PAINT it was 50 ms.^2^ With exposure times of 50–100 ms, PAINT methods using currently available fluorophores, would benefit from using interactions with even higher off‐rates, up to 10–20 s^−1^. On‐rates ideal for PAINT experiments are those that will enable rapid re‐binding of fluorescent probes to molecules of interest. The ideal value will be dictated by the off‐rate of the interaction, the desired *K*_*d*_ of the interaction (which will depend on the circumstances of the imaging) and the concentration of the fluorescently labeled construct the user desires to use.

Although *K*_*d*_ has been reported for many peptide–protein interactions, far fewer on‐ and off‐rates for peptide–protein interactions have been measured. Making more such measurements, and compiling a database of these values, which is accessible to those who wish to develop protein‐based PAINT approaches, would be of great value.

## 6 DISCUSSION

Since its initial implementation in 2006, PAINT‐based SMLM has seen many innovative applications.^1^, ^2^, ^5^, ^7^, ^8^, ^9^, ^10^, ^11^, ^12^, ^13^, ^14^, ^15^, ^16^, ^17^, ^18^, ^19^, ^20^, ^21^, ^22^, ^36^ The development of DNA‐PAINT in 2010 represented a sea change, by providing a means to incorporate high specificity into the transient interaction. DNA‐PAINT has since been optimized and extended in many ways.^9^, ^10^, ^11^, ^12^, ^13^, ^14^, ^15^, ^16^, ^17^, ^18^, ^19^, ^20^, ^21^, ^22^

Recent work, which is the focus of this review, has demonstrated the potential for using peptide–protein interactions, rather than DNA duplex formation, to achieve transient labeling. Peptide–protein interactions are less straightforward to design than a complementary DNA duplex. Nevertheless, they offer significant advantages over using DNA. The binding/unbinding rates can be faster and, most importantly, the peptide–protein pair can be genetically encoded and the complete PAINT methodology achieved within live cells.

What additional features could be incorporated to extend the use of PAINT using peptides and proteins inside live cells?

The ability to tag and image several different cellular targets at the same time, which requires several orthogonal peptide–protein pairs along with several different colored, monomeric, fluorescent proteins would be a significant advance. Novel strategies to image very high or very low abundance cellular proteins, taking advantage of the ability to reduce expression levels of the labeling protein or to collect data for extended periods, would be a welcome addition to the LIVE‐PAINT repertoire. Expansion of the LIVE‐PAINT technology for use within live mammalian cells is also possible using a variety of genome engineering strategies.

The recent work using peptide–protein interactions for PAINT imaging has also revealed how little detailed kinetic information is available for peptide–protein interactions. In order to further develop peptide and protein‐based PAINT methods, many orthogonal peptide–protein interactions pairs with well‐characterized binding kinetics are needed. In addition, a predictive understanding of how mutations can change both on‐ and off‐rates of an interaction would be extremely useful.

An interesting example of a mutation which has a large effect on ligand binding kinetics, but a lesser effect on *K*_*d*_ is seen with carbonic anhydrase. Huang et al. reported that removal of a H‐bonding secondary interaction to a zinc‐liganding His at the active site of carbonic anhydrase increased the off rate for zinc by a factor of 10^6^, but increased the *K*_*d*_ by a factor of only 10^3^.^37^ Presumably, therefore, the on‐rate was also increased by a factor of 10^3^. This result suggests it is possible to mutate proteins in such a way that on‐rates and off‐rates are preferentially affected compared to the overall *K*_*d*_. While this is admittedly a challenging engineering task, it would greatly improve the protein synthetic biology toolbox to have a set of interaction pairs which have similar *K*_*d*_s and but which differ in their on‐ and off‐rates.

In summary, peptide and protein‐based PAINT, and LIVE‐PAINT in particular, present exciting opportunities for enhanced visualization of cellular processes and for protein scientists to apply their expertise to devise even better interaction pairs for such experiments.

## AUTHOR CONTRIBUTIONS

**Curran Oi:** Conceptualization; writing‐original draft; writing‐review and editing. **Simon G. J. Mochrie:** Funding acquisition; writing‐review and editing. **Mathew H. Horrocks:** Funding acquisition; writing‐review and editing. **Lynne Regan:** Conceptualization; funding acquisition; writing‐original draft; writing‐review and editing.

## CONFLICT OF INTEREST

The authors declare no potential conflict of interest.

## References

[1] Eklund AS , Ganji M , Gavins G , Seitz O , Jungmann R . Peptide‐PAINT super‐resolution imaging using transient coiled coil interactions. Nano Lett. 2020;20:6732–6737. 32787168 10.1021/acs.nanolett.0c02620 PMC7496730

[2] Oi C , Gidden Z , Holyoake L , et al. LIVE‐PAINT allows super‐resolution microscopy inside living cells using reversible peptide‐protein interactions. Commun Biol. 2020;3:458. 32820217 10.1038/s42003-020-01188-6 PMC7441314

[3] Perfilov MM , Gurskaya NG , Serebrovskaya EO , et al. Highly photostable fluorescent labeling of proteins in live cells using exchangeable coiled coils heterodimerization. Cell Mol Life Sci. 2020;31894363. 10.1007/s00018-019-03426-5 PMC7329588 31894363

[4] Sharonov A , Hochstrasser RM . Wide‐field subdiffraction imaging by accumulated binding of diffusing probes. Proc Natl Acad Sci U S A. 2006;103:18911–18916. 17142314 10.1073/pnas.0609643104 PMC1748151

[5] Bongiovanni MN , Godet J , Horrocks MH , et al. Multi‐dimensional super‐resolution imaging enables surface hydrophobicity mapping. Nat Commun. 2016;7:13544. 27929085 10.1038/ncomms13544 PMC5155161

[6] Horrocks M , Lee S , Klenerman D . Diagnosis and treatment of neurodegenerative disorders Patent WO2016001644; 2015.

[7] Horrocks MH , Lee SF , Gandhi S , et al. Single‐molecule imaging of individual amyloid protein aggregates in human biofluids. ACS Chem Nerosci. 2016;7:399–406. 10.1021/acschemneuro.5b00324 PMC4800427 26800462

[8] Whiten DR , Zuo Y , Calo L , et al. Nanoscopic characterisation of individual endogenous protein aggregates in human neuronal cells. Chembiochem. 2018;19:2033–2038. 30051958 10.1002/cbic.201800209 PMC6220870

[9] Jungmann R , Steinhauer C , Scheible M , Kuzyk A , Tinnefeld P , Simmel FC . Single‐molecule kinetics and super‐resolution microscopy by fluorescence imaging of transient binding on DNA origami. Nano Lett. 2010;10:4756–4761. 20957983 10.1021/nl103427w

[10] Agasti SS , Wang Y , Schueder F , Sukumar A , Jungmann R , Yin P . DNA‐barcoded labeling probes for highly multiplexed exchange‐PAINT imaging. Chem Sci. 2017;8:3080–3091. 28451377 10.1039/c6sc05420j PMC5380918

[11] Baker MAB , Nieves DJ , Hilzenrat G , Berengut JF , Gaus K , Lee LK . Stoichiometric quantification of spatially dense assemblies with qPAINT. Nanoscale. 2019;11:12460–12464. 31120079 10.1039/c9nr00472f

[12] Dai M . DNA‐PAINT super‐resolution imaging for nucleic acid nanostructures. Methods Mol Biol. 2017;1500:185–202. 27813009 10.1007/978-1-4939-6454-3_13

[13] Dai M , Jungmann R , Yin P . Optical imaging of individual biomolecules in densely packed clusters. Nat Nanotechnol. 2016;11:798–807. 27376244 10.1038/nnano.2016.95 PMC5014615

[14] Jungmann R , Avendano MS , Dai M , et al. Quantitative super‐resolution imaging with qPAINT. Nat Methods. 2016;13:439–442. 27018580 10.1038/nmeth.3804 PMC4941813

[15] Jungmann R , Avendano MS , Woehrstein JB , Dai M , Shih WM , Yin P . Multiplexed 3D cellular super‐resolution imaging with DNA‐PAINT and Exchange‐PAINT. Nat Methods. 2014;11:313–318. 24487583 10.1038/nmeth.2835 PMC4153392

[16] Nieves DJ , Gaus K , Baker MAB . DNA‐based super‐resolution microscopy: DNA‐PAINT. Genes. 2018;9:621. 10.3390/genes9120621 PMC6315775 30544986

[17] Schnitzbauer J , Strauss MT , Schlichthaerle T , Schueder F , Jungmann R . Super‐resolution microscopy with DNA‐PAINT. Nat Protoc. 2017;12:1198–1228. 28518172 10.1038/nprot.2017.024

[18] Schueder F , Lara‐Gutiérrez J , Beliveau BJ , et al. Multiplexed 3D super‐resolution imaging of whole cells using spinning disk confocal microscopy and DNA‐PAINT. Nat Commun. 2017;8:2090. 29233999 10.1038/s41467-017-02028-8 PMC5727263

[19] Strauss S , Jungmann R . Up to 100‐fold speed‐up and multiplexing in optimized DNA‐PAINT. Nat Methods. 2020;17:789–791. 32601424 10.1038/s41592-020-0869-x PMC7610413

[20] Wade OK , Woehrstein JB , Nickels PC , et al. 124‐color super‐resolution imaging by engineering DNA‐PAINT blinking kinetics. Nano Lett. 2019;19:2641–2646. 30864449 10.1021/acs.nanolett.9b00508 PMC6463241

[21] Schlichthaerle T , Eklund AS , Schueder F , et al. Site‐specific labeling of affimers for DNA‐PAINT microscopy. Angew Chem Int Ed Engl. 2018;57:11060–11063. 29873161 10.1002/anie.201804020

[22] Strauss S , Nickels PC , Strauss MT , et al. Modified aptamers enable quantitative sub‐10‐nm cellular DNA‐PAINT imaging. Nat Methods. 2018;15:685–688. 30127504 10.1038/s41592-018-0105-0 PMC6345375

[23] Vijayakumar M , Wong K‐Y , Schreiber G , Fersht AR , Szabo A , Zhou H‐X . Electrostatic enhancement of diffusion‐controlled protein‐protein association: Comparison of theory and experiment on barnase and barstar. J Mol Biol. 1998;278:1015–1024. 9600858 10.1006/jmbi.1998.1747

[24] Cohen‐Khait R , Schreiber GA‐O . Selecting for fast protein‐protein association as demonstrated on a random TEM1 yeast library binding BLIP. Biochemistry. 2018;57:4644–4650. 29671590 10.1021/acs.biochem.8b00172

[25] Phillip Y , Kiss V , Schreiber G . Protein‐binding dynamics imaged in a living cell. Proc Natl Acad Sci U S A. 2012;109:1461–1466. 22307600 10.1073/pnas.1112171109 PMC3277120

[26] Chao H , Houston ME , Grothe S , et al. Kinetic study on the formation of a de novo designed heterodimeric coiled‐coil: Use of surface plasmon resonance to monitor the association and dissociation of polypeptide chains. Biochemistry. 1996;35:12175–12185. 8810925 10.1021/bi9530604

[27] Litowski JR , Hodges RS . Designing heterodimeric two‐stranded α‐helical coiled‐coils: Effects of hydrophobicity and α‐helical propensity on protein folding, stability, and specificity. J Biol Chem. 2002;277:37272–37279. 12138097 10.1074/jbc.M204257200

[28] Gröger K , Gavins G , Seitz O . Strand displacement in coiled‐coil structures: Controlled induction and reversal of proximity. Angew Chem Intl Ed. 2017;56:14217–14221. 10.1002/anie.201705339 28913864

[29] Mikhaylova M , Cloin BMC , Finan K , et al. Resolving bundled microtubules using anti‐tubulin nanobodies. Nat Commun. 2015;6:7933–7933. 26260773 10.1038/ncomms8933 PMC4918323

[30] D'Andrea LD , Regan L . TPR proteins: The versatile helix. Trends Biochem Sci. 2003;28:655–662. 14659697 10.1016/j.tibs.2003.10.007

[31] Pratt SE , Speltz EB , Mochrie SGJ , Regan L . Designed proteins as novel imaging reagents in living *Escherichia coli* . Chembiochem. 2016;17:1652–1657. 27304706 10.1002/cbic.201600252

[32] Hinrichsen M , Lenz M , Edwards JM , et al. A new method for post‐translationally labeling proteins in live cells for fluorescence imaging and tracking. Protein Eng Des Sel. 2017;30:771–780. 29228311 10.1093/protein/gzx059 PMC6680098

[33] Thompson KE , Bashor CJ , Lim WA , Keating AE . SYNZIP protein interaction toolbox: in vitro and in vivo specifications of heterospecific coiled‐coil interaction domains. ACS Synth Biol. 2012;1:118–129. 22558529 10.1021/sb200015u PMC3339576

[34] Speltz EB , Nathan A , Regan L . Design of protein‐peptide interaction modules for assembling supramolecular structures in vivo and in vitro. ACS Chem Biol. 2015;10:2108–2115. 26131725 10.1021/acschembio.5b00415

[35] Thomas F , Boyle AL , Burton AJ , Woolfson DN . A set of de novo designed parallel heterodimeric coiled coils with quantified dissociation constants in the micromolar to sub‐nanomolar regime. J Am Chem Soc. 2013;135:5161–5166. 23477407 10.1021/ja312310g

[36] Lee J‐E , Sang JC , Rodrigues M , et al. Mapping surface hydrophobicity of α‐synuclein oligomers at the nanoscale. Nano Lett. 2018;18:7494–7501. 30380895 10.1021/acs.nanolett.8b02916 PMC6295917

[37] Huang C‐C , Lesburg CA , Kiefer LL , Fierke CA , Christianson DW . Reversal of the hydrogen bond to zinc ligand histidine‐119 dramatically diminishes catalysis and enhances metal equilibration kinetics in carbonic anhydrase II. Biochemistry. 1996;35:3439–3446. 8639494 10.1021/bi9526692

